# Crop Improvement: Where Are We Now?

**DOI:** 10.3390/biology11101373

**Published:** 2022-09-20

**Authors:** Pierre Sourdille, Pierre Devaux

**Affiliations:** 1 UMR 1095 Genetics, Diversity and Ecophysiology of Cereals, 5, INRAE–Université Clermont-Auvergne, Chemin de Beaulieu, 63000 Clermont-Ferrand, France; 2 Florimond Desprez—Recherche & Innovation, 3 Rue Florimond Desprez, PO Box 41, 59242 Cappelle-en-Pévèle, France

Improving the production of all crops is crucial to meeting the challenge of the growing needs related to the simultaneous increase in the world population and demands from farmers and end-users. Moreover, these improvements will have to be developed in the context of sustainable agriculture less demanding in pesticides, insecticides, fertilizers and water, while protecting the environment and biodiversity. In addition, while yield has been the main trait that has been focused on during the second half of the 20th century, this will be no longer the case. Along with high yields, new ideotypes must show good resistance to diseases and pests, tolerance to more extreme weather conditions with extended periods of drought and high temperatures, and at the same time maintain or increase nutritional value.

These requirements hold true for most of the 250 species that have gone through domestication and breeding, although differences exist between crops regarding the best ways to achieve these goals [1,2]. In this Biology Special Issue “Crop Improvement: Now and Beyond”, we want to take stock of recent advances in plant breeding technologies relevant to the challenges of producing more and better [2]. This was also an opportunity to give an indication to readers as to how researchers tackle the various aspects of selection in different crops (monocots or dicots, perennial or annual) and the new technologies available to achieve these goals. This issue contains 23 manuscripts related to eight species (wheat, eggplant, *Brassica rapa*, *Brassica napus*, potato, *Gosypium hirsutum*, sugar beet, and pigeon pea) and contains several comprehensive reviews on the new approaches that are currently used (or soon be deployed) in breeding programmes including the most up-to-date molecular techniques involving epigenetics.

Improving our crop varieties to meet the challenges of increasing crop yields in a sustainable manner in the face of environmental variations resulting from climate changes is a daunting task. Clearly, the best way to achieve this is to coordinate work in laboratories with complementary expertise, simultaneously including a wide range of crops. Such an initiative gathering more than 60 universities and research institutes from 17 EU member states was initiated in 2016 by Wageningen University & Research. This resulted in the creation of a Coordination and Support Action (CSA) called “CropBooster-Preparatory” (CropBooster-P; H2020, GA 817690) in 2018 [3]. The final roadmap will be finalized in 2022 as the “The CropBooster Program”. This large European project will focus its actions on (1) the scientific and technical improvement of crop varieties; (2) the environmental, social, and economic impacts of the proposed improvements; and (3) societal acceptance.

A similar initiative was launched in France in 2011 with a large public–private partnership (BREEDWHEAT project; ANR-10-BTBR-03 [4]) bringing together 26 public and private research groups over 10 years. It was designed to address the challenges of (i) the world-wide structure of wheat genetic diversity; (ii) understanding the factors underlying the traits of importance; (iii) the development of new original approaches to improve breeding; and (iv) providing the breeders and the international wheat community with all the necessary information to develop new powerful ideotypes. The results obtained during this project, and made available to the community, will pave the way for the introduction of the most recent breeding tools (SNP arrays, high-throughput phenotyping, selection models…) to design less vulnerable and more resilient agricultural systems that can meet the challenge of higher wheat production in a sustainable and fluctuating agricultural context and make optimal use of available wheat genetic diversity.

One way to efficiently develop well-adapted varieties for any crop is to explore the wide reservoir of crop wild relatives (CWR; [5]). In their extensive review, Sharma et al. ([6]) highlighted the fundamental role of the International Gene Banks that store and curate wheat landraces as well as wild relatives that may serve as potential sources of new genetic diversity. They provided a reminder of wheat evolution and domestication history and an overview of the botanical classification of wheat and *Aegilops* species including their ploidy status and genome formula. They also reviewed how genetic resources have been used in recent decades to introduce useful alleles to improve wheat varieties. This gives breeders routes to follow to better explore and exploit the wheat genepool. They proposed developing an original stepwise approach to efficiently pre-breed new valuable progenitors that could further be used as parents in classical programmes.

The same holds true for potato (*Solanum tuberosum* L.) for which the evolution of 1219 potato varieties from different groups (from the years 1800 to 2021 and from five continents (North and South America, Oceania, Asia, Africa, Europe)) was evaluated using a set of 35 Simple Sequence Repeats yielding 407 different alleles [7]. The results show that a limited number of lineages were introduced from the Andes into Europe in the 16th century. However, diversity has been maintained through the reintroduction events into the US in the mid-1800s, leading to increased diversity in the genepool of modern varieties. Moreover, the extensive use of exotic germplasm in recent breeding programmes has resulted in the emergence of new genetic groups. Due to this ancient approach using landraces in potato breeding schemes, the diversity in this species has remained intact with numerous interesting resources for improvement.

Screening and exploiting wild diversity are of particular value for disease resistance [6]. It is a particular feature that resistance genes often cluster together and that several genes for various diseases can be introduced at the same time from the same source. In their study, Fedak et al. [8] introduced into wheat lines multiple resistance genes to fusarium head blight (FHB), leaf rust, stem rust, and stripe rust using diploid (*T. monococcum*), tetraploid (*T. carthlicum* and *T. timopheevi*), and hexaploid (*T. miguschovae*) wild relatives. Genetic mapping of the resistance genes revealed that some chromosomes (5A, 6B) carry clusters of genes. Some lines derived from crosses between this original material and elite lines brought together resistance for up to four different diseases.

Similarly, two more precise examples of the introduction of resistance genes from wild species are given. First, resistance to pod borer (*Helicoverpa armigera*) in pigeon pea (*Cajanus cajan* (L.) Millsp.) was introduced from several accessions of wild *Cajanus* species (*C. scarabaeoides* and *C. acutifolius*) with strong resistance, and different resistance mechanisms (antixenosis and antibiosis; [9]). More than 2300 lines incorporating introgressions were produced, with 21 showing a good tolerance to pod borer that may serve as a source of resistance suitable for use in breeding programs. Secondly, resistance to early blight caused by *Alternaria solani* was introduced in cultivated potato from two wild species [10]. *Solanum berthaultii* brought a classical quantitative resistance while interestingly and unexpectedly, the cross with *S. commersonii* ssp. *malmeanum* led to a triploid progeny in which resistance was inherited dominantly. Both results are promising for the development of varieties with new and original natural resistance, meeting the challenge of sustainable production.

The introduction of new diversity from exotic cultivars or wild relatives relies on meiotic recombination (also known as crossover; CO), a biological process common to all eukaryotes with sexual reproduction including angiosperms (for review see [11]). CO is a large reciprocal exchange of DNA segments that occurs between homologous chromosomes originating from the female and the male parents. The occurrence of CO is highly controlled. One CO is mandatory to ensure faithful segregation of chromosomes but there are usually no more than three COs per chromosome pair per meiosis [11]. COs are not evenly distributed along the chromosomes. In species with huge chromosomes such as bread wheat, they mainly cluster distally in the sub-telomeric regions, which prevents allele reshuffling in the pericentromeric regions [12]. In allopolyploid species such as bread wheat or oilseed rape (*Brassica napus*), recombination between highly similar but not homologous (called therefore homoeologous), related chromosomes, is also highly controlled, preventing easy introduction of genes derived from wild relatives [13].

In their review, Fayos et al. [14] surveyed the recent advances in the understanding of recombination manipulation in terms of increasing (having more than one CO per chromosome arm) as well as in distribution (recombination in CO-depleted regions). After an extensive review of the mechanisms underlying the control of meiotic recombination as well as the factors that affect its distribution (sequence features, epigenetic landmarks), they proposed an overview of the various factors that contribute to its stimulation and/or redistribution. These include a modulation by environmental conditions (temperature) or the reduction in the expression of genes involved in recombination control (*RecQ4*, *FancM*, *Figl1* [15]). They opened promising original perspectives for using the site-specific properties of genome-editing technologies (CRISPR/Cas9) to target meiotic recombination at specific chromosomal regions, which would allow the breaking of tight linkage between genes of agronomical interest and undesirable traits.

One original example of increased recombination comes from allo-triploid hybrids derived from the cross between turnip (*Brassica rapa*; 2n = 2x = 20; AA genome) and oilseed rape (*Brassica napus*; 2n = 4x = 38; AACC genome). In this case, crossover events are 3.6 times more numerous on the A chromosomes of the allo-triploid (AAC) than on exactly the same A chromosomes of either the diploid (AA) or the tetraploid (AACC) [16]. Moreover, the number of crossovers is increased in all chromosome regions, including usually CO-poor regions. The authors demonstrated that numerous (21 per plant on average) small (7.6 Mb on average) introgressions from the A genome of *B. rapa* occurred throughout the A genome of *B. napus*. This resulted in the introduction of a small region conferring resistance to *Leptosphaeria maculans*, one of the major pathogens of oilseed rape. This triploid approach has proven useful for the efficient introduction of new variability derived from related species.

Usually, recombination between related homoeologous chromosomes such as those of *B. rapa* and *B. napus* is highly controlled in oilseed rape through the effect of a major locus called *PrBn* (for Pairing regulator in *Brassica napus*; [17]). Similarly, recombination between wheat homoeologous chromosomes is prevented by the presence of a major locus named *Ph1* (for *Pairing homoeologous 1*) located on the long arm of chromosome 5B [18]. It was recently shown that the main gene controlling homoeologous recombination from this locus was *TaZip4-B2* [19]. A CRISPR/Cas9 mutant of *TaZip4-B2* was evaluated for pollen viability and grain setting [20]. This mutant has a 38 amino acid deletion leading to 56% of abnormal meiocytes, which is similar to the *ph1b* mutant. Half (50%) of the meiocytes contained micronuclei. Together, this resulted in a massive loss of fertility (50% reduction of grain number). However, the various *TaZip4-B2* mutants will undoubtedly be useful for the introgression of new diversity from wild species into the wheat genome.

Breeding new varieties relying on classical crosses and recombination pathways is a long and tedious process that can take up to ten years before the release of derived cultivars. However, new technological innovations can significantly shorten the generation time of varieties and increase genetic gain. One of the best examples is the use of haplo-diploidisation (or doubled haploids; DHs) that permits the production of fully homozygous varieties within one generation. Production of DH lines has been achieved for hundreds of species, some being more or less recalcitrant to this process [21]. Among these is eggplant (*Solanum melongena* L.), a species with growing economic interest. In this review [22], the authors surveyed different protocols allowing the production of eggplant DH lines, including anther and microspore culture. They underlined the critical role of genotype in determining the embryogenic response of microspores. They opened new perspectives for the future development of DH production in eggplant including the screening of mutants of the centromeric histone H3 (*CENH3*) gene to generate haploid-inducer lines. Producing more fully homozygous lines will boost the development and commercialization of more productive and more resilient hybrids.

Hybrids are usually found to perform better than pure lines. This is the case with maize (*Zea mays* L.), for which yields have increased 5.5-fold during the last 75 years [23]. Hybrids are difficult to produce in wheat because its self-fertility requires either the use of chemical hybridizing agents or genetic/molecular systems [24,25] to ensure hybrid production and because of a low rate of cross pollination. To improve the production of wheat hybrids, Gimenez et al. [26] used cytoplasmic male sterility (CMS) to produce 91 hybrids that were evaluated for their agronomical performances in three locations. The grain filling phase was longer in hybrids and senescence was delayed resulting in a 7.7% increase in thousand kernel weight. Unexpectedly, the protein content was improved in hybrids by 4.7%. Despite the difficulty of producing hybrids in wheat, the data also showed the potential for higher yields with lower fertilizer and pesticide input.

Among the other biotechnological approaches to rapidly produce new cultivars is the introduction of the relevant genes using transgenesis or genome editing. Three naturally occurring resistance genes to potato late-blight disease (caused, by *Phytophthora infestans*) were introduced in the variety Victoria [27] and the resulting transgenic events were evaluated in field conditions over three years for their agronomical performances, including late-blight resistance. One line (Vic. 172) was completely resistant, with an agronomic performance not statistically different from the Victoria control. This shows the great potential of transgenic plants to underpin the development of new and powerful cultivars.

Another strategy to reduce the duration of the development of varieties is to significantly improve the seed-to-seed cycle, which remains one of the main bottlenecks in plant breeding [28]. Speed breeding aims to increase the number of generations per year, leading up to a three-fold improvement. This approach has been implemented for both short-day and long-day species. Speed breeding relies on the cultivation of plants in highly controlled environments, especially with regards to photoperiod extension and temperature. This requires often expensive high-standard facilities (greenhouses, growth chambers), which currently hampers its widespread application across diverse crops and in developing countries. Efforts to correlate speed breeding with high-throughput phenotyping requires care to avoid any bias that may result from such controlled conditions in order to improve the rate of gain in the production of new original cultivars.

Phenotyping is also central to the development of new varieties. Disease resistance is often difficult to evaluate in the field where the population of pathogens are diverse with several diseases and multiple strains of each. Moreover, the environment may play a crucial role in symptom development. However, if resistance quantification performed in controlled conditions is to be of value, there must be a strong correlation between these data and true resistance in the field. In their study, Beukert et al. [29] reported an approach aiming at identifying seedling leaf-rust resistance in European wheat elite lines, combining detached-leaf assay with high throughput screening with a robot (Macrobot platform). Even if correlation remained moderate (0.38–0.45) between in vitro assays and adult plant resistance, the results are promising and there is room to improve this approach, especially with regards to the diversification of rust strains.

Finally, Genomic Selection (GS) has the power to accelerate the breeding of new cultivars since the selection of the best lines can be predicted using genotype data and a suitable precalculated prediction model thus improving the cost/benefit of breeding programs. If GS was proven useful in cattle breeding [30], things are more complicated in plants where the Genotype x Environment (GxE) interactions play a central role [31]. As a quantitatively inherited trait controlled by several genes with minor effect, Fusarium head blight (FHB) resistance seems a good model to evaluate the factors that affect accuracy prediction of GS in wheat [32]. Using a multi-parental (one resistant parent crossed by three susceptible lines) population of 580 DH lines genotyped with 4205 SNPs, it was found that larger sample sizes of the training population (150 individuals) contribute to an improved accuracy in the GS of FHB resistance. When a major locus exists, fixing its effect in the model improves the prediction accuracy of >0.1 for most of the FHB-related traits. The best prediction is achieved when the training population is closely related to the lines being evaluated. Therefore, these results seem promising for the application of GS in wheat.

The efficiency of marker-assisted selection (MAS) also relies on strong association between markers and traits (or even the genes involved). Restriction-site associated DNA sequencing (RAD-Seq) was performed on 102 sugar beet genotypes showing resistance or susceptibility to *Rhizoctonia solani* causing root and crown rot [33]. Of roughly 40,000 SNPs analysed, 62 showed a strong association with *Rhizoctonia* resistance. One SNP (RsBv1) located on chromosome 6 showed the strongest association (10% of the variability of the resistance explained) with a C-allele associated with resistance while the T-allele was mainly found in susceptible lines. This marker can therefore be used in marker-assisted breeding for *Rhizoctonia* resistance in sugar beet and can also serve as a starting point for the positional cloning of the underlying resistance gene.

Similarly, MAS was applied to introduce leaf rust resistance in elite Argentinian wheat varieties [34]. In a preliminary study, resistance genes *LrBMP1*, *Lr3*, *Lr16*, and *Lr17* were mapped onto the wheat genome using 94 recombinant inbred lines (RILs) from segregating populations (Buck Manantial (resistant) x Purplestraw (susceptible)) and a set of 530 SSR and AFLP markers. Fine mapping of *LrSV1*, *LrSV2*, *LrcSV2*, and *LrG6* was performed on two large F2 populations using Sinvalocho MA and G6 as resistant parents. The most highly associated markers for all these genes were used to pyramid the resistance into seven wheat cultivars. These varieties showed good resistance to leaf rust in the field and will be suitable for commercial use. Moreover, fine mapping of some of these genes (*LrSV2*, *LrcSV2*) provides a springboard for their further positional cloning.

Though many studies focus their efforts on resistance genes allowing the reduction in use of pesticides and fungicides, others focus on development traits and especially on earliness which may help in the avoidance of heat and drought stresses in crops. This is the case for the genes involved in earliness per-se, in photoperiod response, and vernalization in wheat (respectively *Eps*, *Ppd,* and *Vrn* genes) [35]. These genes affect grain yield since they impact the phenology of the plant. This may shorten the grain-filling stage, resulting in lower yields. Yield can be measured as the quantity of biomass produced per m^2^. Using two association panels, and each of 150 elite spring bread wheat types (including 57 lines common to the two panels and used as controls) evaluated for two years and one location, it was shown that the *Ppd*-insensitive allele *Ppd-D1a* had the most significant positive effect on grain yield in both panels followed by the two *Eps* alleles *TaTOE-B1* and *TaFT3-B1*.

Similarly, in cotton (*Gossypium hirsutum*), some genes of the plant-specific transcription factor family TEOSINTE-BRANCHED1/CYCLOIDEA/PCF (TCP) contribute to fibre elongation, to salt and drought stress responses, to auxin response regulation, and to branching development [36]. One of these transcription factors (*GhTCP62*) is highly expressed in axillary cotton buds. The authors conducted an overexpression assay of *GhTCP62* in Arabidopsis where the amino acid sequence of this protein was highly conserved with that of cotton, suggesting a similar function in the two species. Development of Arabidopsis plants overexpressing *GhTCP62* was strongly affected with significantly reduced branching. Since *GhTCP62* shares a high homology with *GhTCP32* (*GhBRC1*) that regulates the expression levels of *GhHB21* and *GhHB40* (two genes involved in the regulation of bud dormancy), it is speculated that *GhTCP62* could also be involved in the same pathway.

Identifying the right candidate genes or the orthologues that bridge between the related species relies on high-quality and well annotated sequences. In recent years, highly accurate whole-genome sequences can now be easily generated, especially by using long-read sequencing technologies and adapted algorithms for read assembly. In this work, the authors used a combination of long-read sequencing (Oxford Nanopore PromethION sequencer), optical mapping (BioNano Genomics), and Omni-C or Pore-C (Dovetail Genomics, and Oxford Nanopore Technologies, respectively) for long-distance interactions to reconstruct the genome of *Brassica rapa* variety Z1 [37]. The best assembly was achieved when long reads were associated with optical maps and Pore-C long-distance interactions. The new assembly covered a higher proportion of the estimated genome length and the rate of ambiguous bases (N) dropped from 8.22% to 0.66%.

If classical genetic approaches combined with the most advanced technologies enable plant breeding to maintain genetic gains and production, new areas of research are opening up especially with regards to acclimatization and adaptation to climate changes. Among these is epigenetics, which has emerged as an important mechanism for gene regulation and expression. In their extensive review, Kakoulidou et al. [38] briefly introduced the various epigenetic pathways for chromatin signalling (DNA methylation, Histone variants) as well as RNA molecules involved in gene regulation (siRNAs, miRNAs, lncRNA) and then they reviewed the results achieved both in model systems and in translation to crops with regard to natural or induced epigenetic diversity as well as its propagation. Finally, they covered the implementation of molecular approaches to improve our knowledge of this promising topic.

Salinity tolerance in plants is given as an example of the importance of epigenetic variation. This is becoming one of the most harmful abiotic stresses since it leads to physiological and morphological effects, resulting in major yield loss [39]. This review firstly covered the main physiological and biochemical bases of salt tolerance in plants and then summarized the underlying genetic base. The authors proposed different approaches to improve salt tolerance in crops including genetic engineering or genome editing of the most important genes. They illustrated epigenetic modifications (development of epiRILs) that may contribute to the improvement of salt tolerance and concluded by highlighting the importance of the microbiome with regard to the role of *Rhizobacteria* in improving the salt tolerance of crops. 

To conclude, in this Special Issue “Crop Improvement: Now and Beyond”, all contributors underline the challenge that agriculture will have to face in the coming decades. There is a requirement to increase and improve crop production and develop resilient varieties with improved sustainable production in terms of quantity and quality within the context of climate change. They propose a wide range of innovative strategies to enhance cultivar development, adaptable to most annual or perennial species. This includes diversity re-enrichment using crop wild relatives, crossed with elite lines and curation of interspecific hybrids to reduce, or even remove, deleterious traits while maintaining high levels of fertility and productivity. Improving the rate of genetic gain in breeding will also be a challenge but relevant strategies using short multiplication cycles, high-throughput phenotyping, and trait-marker associations will speed-up the process. Moreover, new molecular techniques such as whole-genome sequencing and gene editing will boost our understanding of the factors underlying agronomical traits, and will result in diagnostic makers for them. Finally, the new area of epigenetics will certainly pave the way for the development of more resilient crops, adaptable to a wider range of climates and biotic or abiotic stresses. We have also no doubt that researchers will pursue their efforts in providing breeders with new approaches to follow as well as new powerful tools to exploit. This is why we decided to convert this Special Issue to a Topical Collection that will allow keeping breeders from the crop community informed about the most recent advances for their favourite crop. This will be our contribution to the improvement of production in all crops, promoting more sustainable agriculture (using less pesticides, water, and fertilizers) in the face of the tremendous challenge of abiotic stresses resulting from climate change.
